# Potential Diagnostic Value of the Peripheral Blood Mononuclear Cell Transcriptome From Cattle With Bovine Tuberculosis

**DOI:** 10.3389/fvets.2020.00295

**Published:** 2020-05-27

**Authors:** Lichun Fang, Weidong Lin, Hong Jia, Xintao Gao, Xiukun Sui, Xiaoyu Guo, Shaohua Hou, Yitong Jiang, Liangquan Zhu, Hongfei Zhu, Jiabo Ding, Lin Jiang, Ting Xin

**Affiliations:** ^1^Institute of Animal Sciences (IAS), Chinese Academy of Agricultural Sciences (CAAS), Beijing, China; ^2^Molecular and Cellular Biology, Gembloux Agro-Bio Tech University of Liège (ULg), Gembloux, Belgium; ^3^Department of Inspection Technology Research, China Institute of Veterinary Drugs Control, Beijing, China

**Keywords:** bovie tuberculosis, PBMC, transcriptome, immune response, biomarker, diagnosis

## Abstract

Bovine tuberculosis (bTB) is a chronic disease of cattle caused by *Mycobacterium bovis*. During early-stage infection, *M. bovis*-infected cattle shed mycobacteria through nasal secretions, which can be detected via nested-polymerase chain reaction (PCR) experiments. Little research has focused on immune responses in nested PCR-positive (bTB PCR-P) or nested PCR-negative (bTB PCR-N) *M. bovis*-infected cattle. Here, we investigated the transcriptomes of peripheral blood mononuclear cells (PBMCs), with or without stimulation by purified protein derivative of bovine tuberculin (PPD-B), among bTB PCR-P, bTB PCR-N, and healthy cattle using RNA-Seq. We also explored the potential value of PBMC transcripts as novel biomarkers for diagnosing bTB. Numerous differentially expressed genes were identified following pair-wise comparison of different groups, with or without PPD-B stimulation (adjusted *p* < 0.05). Compared with healthy cattle, bTB PCR-P, and bTB PCR-N cattle shared 5 significantly dysregulated biological pathways, including Cytokine-cytokine receptor interaction, NF-kappa B signaling pathway, Hematopoietic cell lineage, Osteoclast differentiation and HTLV-I infection. Notably, dysregulated biological pathways of bTB PCR-P and bTB PCR-N cattle were associated with cell death and phagocytosis, respectively. Lymphotoxin alpha and interleukin-8 could potentially differentiate *M. bovis*-infected and healthy cattle upon stimulation with PPD-B, with area-under-the-curve (AUC) values of 0.9991 and 0.9343, respectively. B cell lymphoma 2 and chitinase 3-like 1 might enable differentiation between bTB PCR-P and bTB PCR-N upon stimulation with PPD-B, with AUC values of 0.9100 and 0.8893, respectively. Thus, the PBMC transcriptome revealed the immune responses in *M. bovis*-infected cattle (bTB PCR-P and bTB PCR-N) and may provide a novel sight in bTB diagnosis.

## Introduction

Bovine tuberculosis (bTB) is a serious disease in cattle populations that is primarily caused by *Mycobacterium bovis* infection (1, 2). *M. bovis* infects a wide range of hosts including wild animals and humans (3, 4) and is a slow-growing pathogen that causes progressive disease (5). Despite intensive worldwide efforts, bTB remains a huge burden and threat to livestock, with more than 50 million cattle infected worldwide resulting in ~3 billion dollars in economic loss annually (6).

Currently, bTB control and eradication is mainly based on the test-and-slaughter strategy, which involves screening cattle using the tuberculin skin test (TST) alone or in combination with interferon gamma (IFN-γ)-release assay (IGRA). Cattle with positive TST and/or IGRA test results are slaughtered. However, due to the limitations of these tests and the significant economic burden produced by this eradication strategy, *M. bovis* has escaped eradication (7). In humans, based on symptoms, pathology, and microbiological evidence of infection in sputum and/or a nucleic-acid-amplification test (8), tuberculosis (TB) was divided into two states (active and latent infection). However, traditional bTB-diagnosis methods (TST and IGRA) cannot distinguish the stages of bTB progression. Costello reported that at least 15% *M. bovis*-infected cattle shed *Mycobacterium* (9), mainly at an early stage in infection (10). Previously, a nested PCR assay based on *mpb70* was established to detect mycobacteria in nasal exudates and milk (9). Prior research confirmed that about 23.18–87.5% of *M. bovis*-infected cattle were nested PCR-positive (PCR-P) (11, 12). However, few studies have focused on differing immune responses between *M. bovis*-infected cattle determined PCR positive and negative.

Blood transcriptomic profiling has been widely used to examine host-immune responses to infection based on an unbiased analysis and comprehensive overview (13). Recently, in human TB, several blood transcriptomic signatures have been identified to differentiate active TB patients from healthy people (14, 15). A better understanding of the immune response between the proposed states (bTB PCR-P and bTB PCR-N) and earlier screening for disease-specific biomarkers associated with bTB progression would greatly advance bTB diagnostics.

**Figure 1:**
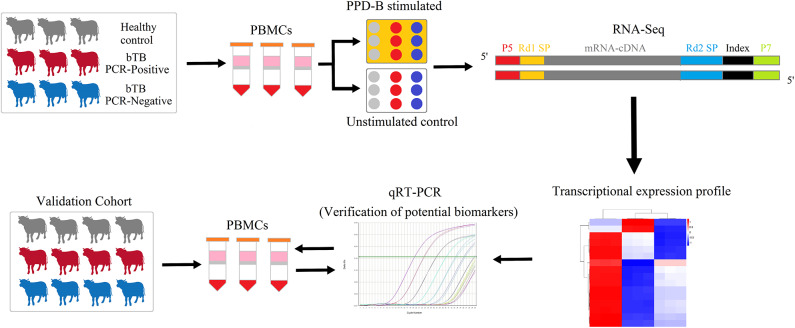
The experimental scheme of the study. Transcriptomes of PPD-B-stimulated and -unstimulated PBMCs from bTB PCR-P, bTB PCR-N, and HC cattle were profiled by RNA-Seq and then were analyzed by bioinformatic methods. Subsequently, the proposed potential biomarkers were validated by qRT-PCR in the validation cohort with 17 bTB PCR-P cattle, 17 bTB PCR-N cattle, and 17 HC cattle.

Here, we investigated peripheral blood mononuclear cell (PBMC) transcriptomes in naturally *M. bovis*-infected cattle and evaluated the diagnostic value of PBMC transcripts. PBMCs isolated from cattle with different infection statuses (i.e., bTB PCR-P, bTB PCR-N, and healthy cattle) were treated with bovine purified protein derivative of bovine tuberculin (PPD-B) or phosphate-buffered saline (PBS) for 6 h. By RNA-Seq, we compared the transcriptomes of PPD-B-stimulated and -unstimulated (PBS-treated) PBMCs between the three groups. Finally, several potential biomarkers were validated for differentiating bTB PCR-P and bTB PCR-N or infected cattle and healthy cattle, as outlined in Figure 1.

## Materials and Methods

### Animals

Holstein cattle (368 total) were chosen from herds with a recent history of *M. bovis* infection (*n* = 243) and that were negative for *M. bovis* (*n* = 125). bTB was detected by TST and IGRA testing, as we described previously (16). Nasal swab samples were analyzed by nested PCR to amplify a specific *M. bovis* fragment to further characterize the infection status. Briefly, a single PCR was run to amplify a segment (372 bp) of the *mpb70* gene with the primers M70F, 5′-GAACAATCCGGAGTTGACAA-3′, and M70R, 5′-AGCACGCTGTCAATCATGTA-3′). Then, a nested PCR was run using 1 μl of the previous reaction mixture to amplify a 208-bp fragment within the 372-bp region of the *mpb70* gene with the primers M22F, 5′-GCTGACGGCTGCACTGTCGGGC-3′, and M22R, 5′-CGTTGGCCGGGCTGGTTTGGCC-3′ (12). Sera from the selected cattle were tested for *M. avium subsp. paratuberculosis*, brucellosis, and bovine viral diarrhea virus antibody production.

### PBMC Preparation, Stimulation, and RNA Extraction

About 50 ml of whole blood with anticoagulant (heparin) was collected aseptically from each animal. PBMCs were isolated using bovine lymphocyte-separation medium (Solarbio, China) by density-gradient centrifugation, per the manufacturer's instructions. Red blood cells (RBC) were removed by RBC lysis, the cells were washed by PBS for three times. And then cells from each animal were counted and seeded into 12-well tissue culture plates at 5 × 106 cells/ml OpTmizer™ T-Cell Expansion SFM (Gibico) containing 1% L-Glutamine (Gibco) and 1% penicillin-streptomycin (Sigma-Aldrich, Ltd.). Subsequently, the PBMCs were stimulated with 300 IU PPD-B (3,000 IU/mL, Prionics AG, Schlieren, Switzerland); unstimulated PBMCs (PBS-treated) from the same cattle were run as a negative control. Subsequently, PBMCs were incubated at 37°C with 5% CO_2_, harvested for treatment 6 h post-stimulation. Harvested cells were washed by ice-cold PBS for three times and then stored at −80°C in TRIzol reagent (Invitrogen, CA, USA) until further use.

Total RNA was extracted using the TRIzol reagent (Invitrogen), following the manufacturer's instructions. The time frame from blood collection to RNA extraction was within 24 h. The quantity and quality of RNA samples were checked using a NanoDrop 2000 spectrophotometer (Thermo Fisher Scientific, Waltham, MA, USA) and an Agilent 2100 Bioanalyzer with the RNA 6000 Nano LabChip Kit (Agilent, Santa Clara, CA, USA). All total RNA samples had an RNA integrity number >7.0, an A260/280 ratio >1.8, and an RNA quantity > 3.0 μg.

### RNA-Seq Library Preparation and Sequencing

A total 18 RNA-Seq labraries were prepared which comprised PPD-B stimulated and unstimulated across 9 cattle in different infection statuses of bTB (three biological replicates: exact 3 *M. bovis*-infected cattle that were nested PCR positive, 3 *M. bovis*-infected cattle that were nested PCR negative and 3 healthy cattle). According to the manufacturer's protocol, the libraries for Illumina sequencing were made with the TruSeq Stranded mRNA sample prep kit (Illumina, San Diego, USA) and sequenced using an Illumina HiSeq 2000 system.

### Bioinformatics Analysis of RNA-Seq Data

For the sake of obtaining high-quality clean reads, the adapter sequence and low-quality sequences were trimmed. All clean reads were mapped to the *Bos taurus* reference genome (UMD3.1.73) using TopHat2 software (17, 18). The data of three biologically repeated samples were assembled by Cuffmerge (19), and the expression levels of genes in different groups were finally obtained. Next, differentially expressed genes (DEGs) were identified using the edgeR algorithms (20). For the RNA-Seq data, genes with expression fold change of >1.5 or < 0.67 and with q-values of <0.05 after correction by false discovery rate (FDR) were considered significant. GO analysis and pathway analysis were performed using the Gene Ontology database (http://geneontology.org/) and Kyoto Encyclopedia of Genes and Genomes database (KEGG) (http://www.genome.jp/Kegg/).

### Validation of RNA-Seq Data by Reverse-Transcription-Quantitative PCR (qRT-PCR)

To validate PBMC mRNA-expression levels observed by RNA-Seq, seven genes in PPD-B-stimulated PBMCs and seven genes in unstimulated PBMCs were randomly selected and simultaneously validated by qRT-PCR. According to the manufacturer' s directions, gDNA was wiped out and the total RNA was reverse transcribed into cDNA with Oligo (dT) primers (Qiagen, Venlo, The Netherlands). Those total RNA samples were the same samples used in RNA-Seq library preparation in section RNA-Seq Library Preparation and Sequencing. For qPCR, triplicate 20 μL reactions containing 10 μL 2 × QuantiNova SYBR Green PCR Master Mix (Qiagen), different pairs of 0.7 μM primers (Supplementary Table 1), and 1 μL of cDNA were analyzed using a CFX96 real-time PCR instrument (Bio-Rad Laboratories, California, USA). An initial 2-min denaturation step at 95°C was followed by 40 PCR cycles (95°C for 5 s and 60°C for 10 s). β-actin mRNA was detected as an endogenous control, and relative gene-expression levels were calculated using the comparative 2^−ΔΔ*Ct*^ method (21). All primers used in this study were designed with Primer Premier 6.0 software and listed in Supplementary Table 1.

### Validation of the Performance of PBMCs Transcripts as Biomarker for bTB Diagnosis

To evaluate the performance of potential biomarkers for bTB, PPD-B-stimulated and unstimulated PBMCs were harvested from 34 naturally *M. bovis*-infection cattle (including 17 bTB PCR-P and 17 bTB PCR-N cattle) and 17 healthy cattle. The RNAs were extracted to measure gene-expression levels using the 2^−ΔΔ*CT*^ method by qRT-PCR, with unstimulated PBMCs serving as a calibrator, and β-actin serving as the reference gene (the primers were shown in Supplementary Table 1). Receiver operating characteristic (ROC) analysis was used to evaluate the diagnostic value of potential biomarkers of interest.

### Statistical Analysis

Data analysis were conducted and visualized by ANOVA followed by Tukey's multiple comparison test using Prism v5.0 (GraphPad Software, La Jolla, CA, USA). *P* < 0.05 were considered significant.

## Results

### Sample Screening and Utilization

Screening results indicated that, of the 368 cattle, 138 cattle were positive for TST, 106 cattle were positive for IGRA, and 32 cattle were positive for nested PCR. Based on the TST, IGRA, nested-PCR and the serological diagnosis results (negative for BVD, Brucella, etc.), we selected 20 naturally *M. bovis*-infected bTB PCR-P and 20 bTB PCR-N cattle (a total of 40 naturally *M. bovis*-infected cattle), as well as 20 age- and sex-matched healthy cattle (HC) for this study. Three cattle in each group were used for RNA-Seq, and the remaining 17 cattle were studied in biomarker-verification experiments.

### Landscape of Differential Gene Expression in Bovine PBMCs

PBMC mRNA-expression levels in cattle in the bTB PCR-P, bTB PCR-N, and HC groups were investigated by high-throughput sequencing. Each sample was divided into two treatment groups, where one was stimulated with PPD-B and the other served as an unstimulated control. Six groups of differentially expressed genes (DEGs) were identified based on our screening criteria (fold-change >1.5 or <0.67; *q* < 0.05) (all of the DEGs are listed in Supplementary Tables 2, 3).

**Figure 2:**
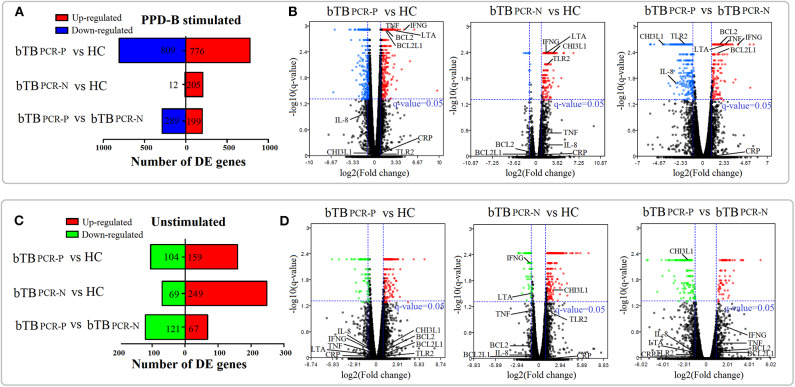
Landscape of DEGs in PPD-B-stimulated and -unstimulated PBMCs. Based on the pair-wise comparisons of bTB PCR-P, bTB PCR-N, and HC group **(A,C)** the number of DEGs in PPD-B-stimulated PBMCs **(A)** and unstimulated PBMCs **(C)** were summarized, and **(B,D)** Volcano plots for difference in gene expression in PPD-B stimulated PBMCs **(B)** and unstimulated PBMCs **(D)** were also drawn. The potential biomarkers are also visualized in the volcano plots.

In the unstimulated treatment groups, compared to the HC cattle, we identified 263 DEGs (159 upregulated and 104 downregulated) and 318 DEGs (249 upregulated and 69 downregulated) in bTB PCR-P and bTB PCR-N cattle, respectively. A total of 188 DEGs (67 upregulated and 121 downregulated) were identified between bTB PCR-P and bTB PCR-N cattle (Figure 2C). Even though the cattle selected for this study were confirmed to be free of usual epidemic diseases (i.e., paratuberculosis, brucellosis, and bovine viral diarrhea), we could not exclude the possibility of infection with other pathogens. Therefore, we used an *M. bovis*-specific antigen (PPD-B) as a stimulus to analyze the adaptive immune responses of *M. bovis*-infected cattle with different infection statuses. Interestingly, in the PPD-B-stimulated groups, a relatively large number of DEGs was observed. Compared to the HC cattle, we identified 1585 DEGs (776 upregulated and 809 downregulated) and 217 DEGs (205 upregulated and 12 downregulated) in bTB PCR-P and bTB PCR-N cattle, respectively. We also identified 488 DEGs (199 upregulated and 289 downregulated) between bTB PCR-P and bTB PCR-N cattle (Figure 2A).

Overall, our current results revealed numerous DEGs in the pair-wise comparisons of different groups with and without stimulated. Volcano plots were also drawn (Figures 2B,D). With this approach, we visualized the expression levels and statistical significances of all genes between the bTB PCR-P, bTB PCR-N, and HC groups.

### Validation of RNA-Seq Data by qRT-PCR

To validate the RNA-Seq data, 14 genes were randomly selected for qRT-PCR analysis. Expression of the interferon-induced protein with tetratricopeptide repeats 1 (*IFIT1*) gene was not detected in unstimulated PBMCs from bTB PCR-N cattle by qRT-PCR because of the low expression level. Our results showed that the expression of most (13/14) of the selected genes was consistent with the log2 (fold-change) values determined by RNA-Seq and qRT-PCR (Table 1).

**Table 1:** Transcriptional level of 14 randomly selected genes by RNA-seq and qRT-PCR.

**Gene**	**PPD-B stimulated PBMCs (Log**_****2****_ **FC)**				**Gene**	**Unstimulated PBMCs (Log**_****2****_ **FC)**			
	**RNA-Seq**		**qPCR**			**RNA-Seq**		**qRT-PCR**	
	**bTB PCR-P/NC**	**bTB PCR-N/NC**	**bTB PCR-P/NC**	**bTB PCR-N/NC**		**bTB PCR-P/NC**	**bTB PCR-N/NC**	**bTB PCR-P/NC**	**bTB PCR-N/NC**
*TIMP1*	−0.872	1.596	−0.982	1.129	*CD69*	−2.196	−1.836	−1.948	−1.776
*TNFAIP2*	0.861	1.845	1.374	1.828	*IFIT1*	−1.062	−1.457	−0.732	–
*THEMIS2*	1.046	2.333	0.988	1.757	*IL17F*	−1.041	−2.527	−0.938	−3.011
*LRP1*	1.049	2.656	1.278	2.225	*IL17*	−2.079	−2.884	−2.413	−2.353
*CYP27B1*	1.067	2.846	2.053	1.810	*THBS1*	2.498	4.515	2.472	3.947
*PSTPIP2*	−0.757	1.249	2.081	2.586	*AMT*	0.967	1.251	1.081	1.725
*SAA1*	−3.743	1.905	−2.372	1.004	*CLCN7*	0.894	1.054	1.028	1.246

–*, not detected by qRT-PCR*.

### PBMC Transcriptomics Elucidated Different Host responses Associated With bTB Progression

To gain specific biological insights into different bTB infection statuses, we analyzed the DEGs using the GO and KEGG databases. To increase the rigor of our analysis, we focused solely on *M. bovis*-specific antigen (PPD-B)-stimulated PBMCs.

**Figure 3:**
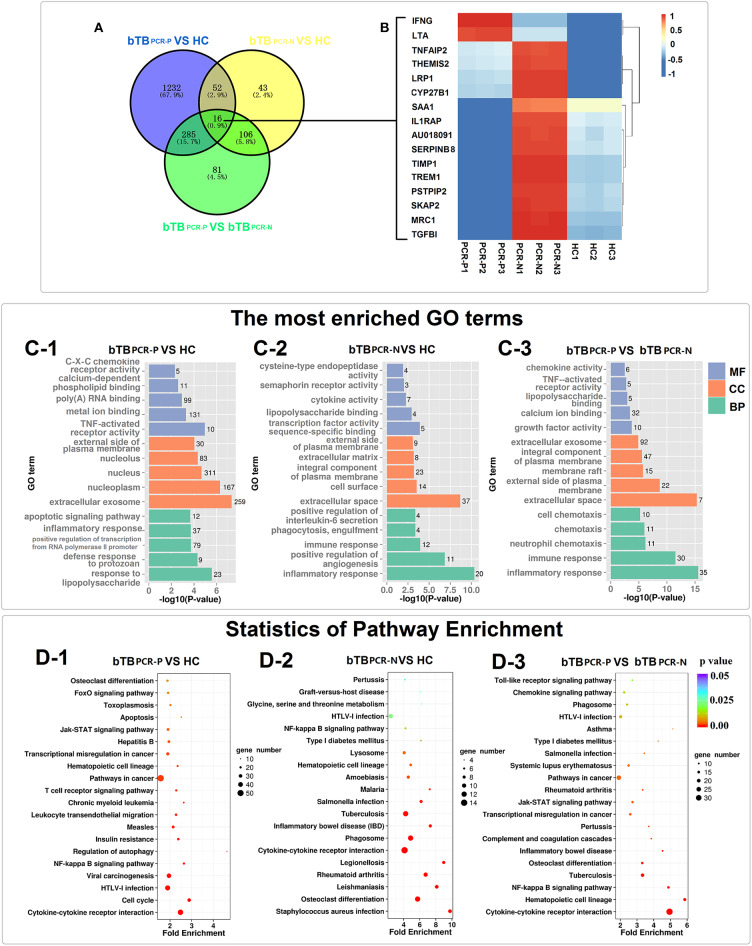
Transcriptome analyses of PPD-B stimulated PBMCs. **(A)** Venn diagrams of DEGs among the three comparing groups. **(B)** Heat map showing the expression of 16 DEGs overlapped in the three comparing groups. The bar indicates relative expression level from high (red) to low (blue). **(C1–3)** The most significantly enriched GO terms of the DEGs in the three comparing groups. GO categories are grouped by biological process (BP), cellular component (CC), and molecular function (MF). **(D1–3)** KEGG pathway analysis of the DEGs among the three comparing groups.

The significantly enriched GO categories (biological process, cellular components, and molecular functions) are summarized in Figure 3C and Supplementary Table 4. GO analysis of bTB PCR-P and HC cattle indicated that the DEGs encode proteins that were mainly located in extracellular exosome and nucleoplasm; possess TNF-activated receptor activity and metal ion binding; and were involved in response to lipopolysaccharide, defense response to protozoan, positive regulation of transcription from RNA polymerase II promoter, inflammatory response and apoptotic signaling pathway (Figure 3C-1). The DEGs between bTB PCR-N and HC cattle encode proteins that were mainly localized in extracellular space and on cell surface; possessed transcription factor activity sequence-specific binding and lipopolysaccharide binding; and were involved in inflammatory response, positive regulation of angiogenesis, immune response, phagocytosis, engulfment, and positive regulation of IL-6 secretion (Figure 3C-2). The DEGs between PCR-positive and -negative bTB cattle encode proteins that were mainly located in extracellular space and external side of plasma membrane; possessed growth factor activity and calcium ion binding; and were involved in inflammatory response, immune response, neutrophil chemotaxis, chemotaxis, and cell chemotaxis (Figure 3C-3).

Given the broad and variable differences in PBMC mRNA-expression levels between healthy and bTB cattle for the PCR-positive and -negative pairs, KEGG analysis was performed to identify dysregulated biological pathways by pooling DEGs into host-gene pathways. The 20 most significantly affected pathways of the three pair-wise comparisons revealed changes in the different biological states (PCR-positive and -negative) of bTB (Figure 3D and Supplementary Table 4). For the most part, the significantly affected pathways were associated with immunobiology. Specifically, five pathways were overlapped in the three comparisons, including Cytokine-cytokine receptor interaction, NF-kappa B signaling pathway, Hematopoietic cell lineage, Osteoclast differentiation and HTLV-I infection (Figures 3D1–3). This result not only indicated that these pathways were dysregulated in bTB cattle but also emphasized the different degrees of dysregulation in bTB PCR-P and bTB PCR-N cattle. In addition, dysregulated pathways associated with cell death were widely identified in bTB PCR-P cattle when compared to HC cattle, including regulation of autophagy, Jak–STAT signaling pathway, apoptosis, and FoxO signaling pathway (Figure 3D–1). The pathways associated with phagocytosis were differentially activated between bTB PCR-N and HC cattle, including phagosomes and lysosomes (Figure 3D–2).

Furthermore, Venn diagram analysis revealed 16 DEGs shared between the bTB PCR-P, bTB PCR-N, and HC groups (Figure 3A). Heatmap analysis showed the relative expression levels of these 16 DEGs in the different groups (Figure 3B).

### Diagnostic Value of PBMC Transcriptional Gene Signatures in bTB Progression

The PBMC transcriptomic profiles observed in this study provided an unbiased, comprehensive overview of host factors that are dysregulated in the progression of bTB infection. Given this phenomenon, we explored the diagnostic value of host PBMC genes in detecting *M. bovis* infection and distinguishing the stage of *M. bovis* infection. Based on the fold-changes, the bioinformatics and Venn diagram analyses of the DEGs, and combined with our previous proteomic results (22), 9 PBMC transcriptional gene signatures were selected as potential biomarkers for further verification (Supplementary Table 5). The expression levels and statistical significances of 9 genes (*IFNG, TNF, IL8, CRP, BCL2L1, BCL2, TLR2, CHI3L1*, and *LTA*) obtained by RNA-Seq were plotted in Figures 2B,D.

**Figure 4:**
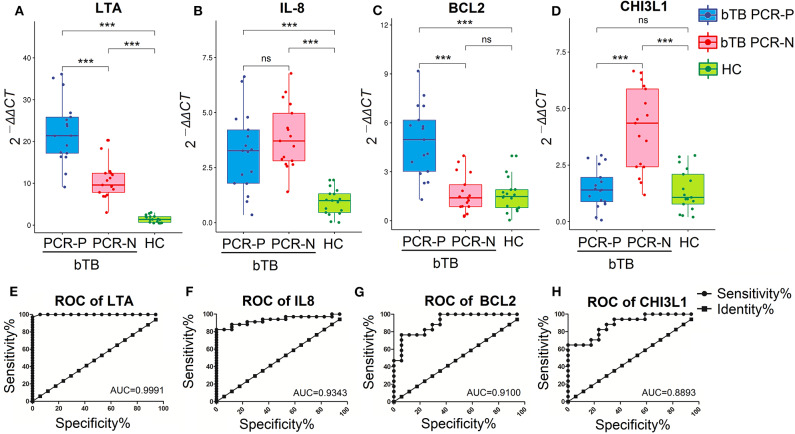
Validation the performance of PBMCs transcripts as biomarker for bTB diagnosis. **(A–D)** The levels of PBMCs transcripts expression in bTB PCR-P (*n* = 17), bTB PCR-N (*n* = 17), and HC cattle (*n* = 17). Expression level of *LTA* **(A)**, *IL-8* **(B)**, *BCL2* **(C)**, and *CHI3L1* **(D)**. Gene expression was calculated using the 2^−ΔΔ*CT*^ method, with unstimulated PBMCs as a calibrator and β-actin as the endogenous control. Dots indicate individual data points, box limits show the 25th and 75th percentiles, center lines indicate the medians, and whiskers extend 1.5 times the interquartile range (IQR). Significant differences were tested using a one-way ANOVA followed by Dunn's multiple-comparison test. ***P < 0.0001. **(E–H)** The diagnostic values of *LTA* **(E)**, *IL-8* **(F)**, *BCL2* **(G)**, and *CHI3L1* **(H)** were analyzed by receiver operating characteristic (ROC) curves.

qRT-PCR and ROC analysis suggested that the *LTA, IL-8, BCL2*, and *CHI3L1* genes serve as valuable, novel biomarkers for bTB diagnosis. The mRNA-expression levels of *LTA* and *IL-8* induced by PPD-B were significantly higher in bTB cattle (including bTB PCR-P and bTB PCR-N) than in HC cattle (*P* < 0.0001; Figures 4A,B), and the AUC values were 0.9991 and 0.9343, respectively (Figures 4E,F). The mRNA-expression level of *BCL2* induced by PPD-B was significantly higher in bTB PCR-P cattle than in bTB PCR-N and HC cattle (Figure 4C), with an AUC value of 0.9100 (Figure 4G). The mRNA-expression level of *CHI3L1* induced by PPD-B was significantly higher in bTB PCR-N cattle than in bTB PCR-P and HC cattle (Figure 4D) with the AUC value of 0.8893 (Figure 4H).

Protein IFN-γ has been used commercially as a valuable biomarker for diagnosing bovine tuberculosis. In this study, the mRNA-expression level of *IFNG* (which encodes IFN-γ) induced by PPD-B was significantly higher in bTB cattle than in HC cattle (Supplementary Figure 1A1), and the AUC value of *IFNG* was 1.0000 (Supplementary Figure 1B1). This result is consistent with those of previous reports. Unfortunately, ROC analysis for *BCL2L1* (AUC = 0.7024) and *TNF-*α (AUC = 0.6851) showed poor diagnostic performance (Supplementary Figures 1B2,B3), although their mRNA-expression levels were higher in bTB cattle than that in HC cattle (Supplementary Figures 1A2,A3). The *TLR2* mRNA-expression level, with or without PPD-B stimulation, was significantly higher in bTB PCR-N cattle than that in bTB PCR-P and HC cattle (data not shown), indicating that it may not be bTB-specific. *CRP* mRNA expression was not high enough to be detected which means it is unable to meet the essential requirements as biomarker.

Collectively, after excluding a known biomarker (*IFNG*) and inappropriate transcripts (*BCL2L1, TNF, TLR2*, and *CRP*), we validated 4 novel potentially reliable biomarkers for bTB diagnosis. PPD-B-stimulated *LTA* and *IL-8* could potentially differentiate *M. bovis*-infected cattle from HC, and PPD-B-stimulated *BCL2* and *CHI3L1* have the potential power in differentiating bTB PCR-P cattle from bTB PCR-N cattle.

## Discussion

World Health Organization pointed out that human TB can only be controlled by eradicating bTB (22). An efficient way to eradicate bTB is to slaughter *M. bovis*-infected cattle, and prioritizing the slaughter of bTB cattle that actively shed mycobacteria was recommended (23). An even more urgent need is to reveal the immune heterogeneity of bTB in different stages, and to develop and apply improved diagnostic techniques for active bTB to prevent them from transmitting bTB.

Using comprehensive PBMC transcriptomics, we showed substantial variability in the immune responses of *M. bovis*-infected cattle (diagnosed by TST and IGRA) determined to be nested-PCR-positive or -negative. We identified 188 and 488 DEGs in unstimulated and PPD-B-stimulated PBMCs between bTB PCR-P and bTB PCR-N cattle, respectively (Figures 2A,C). GO analysis showed different physiological functions between bTB PCR-P and bTB PCR-N cattle. Cellular component ontology displays the classification of DEGs based on the cell structures. Compared to healthy cattle, the DEGs encode proteins in bTB PCR-P cattle mainly located in nucleus (311 genes) and extracellular exosome (259 genes) (Supplementary Table 4 and Figure 3C–1). It is noteworthy that the genes classified in the nucleus which associated with apoptosis were significantly downregulated (Supplementary Table 3). For example, *TP53INP1* (tumor protein p53 inducible nuclear protein 1) and *TPRKB* (TP53RK binding protein) were significantly downregulated which downregulated p53-mediated apoptosis that suppressed cell death (24). The DEGs encode proteins in bTB PCR-N cattle mainly located in the extracellular space (37 genes) (Supplementary Table 4 and Figure 3C–2). These DEGs mainly associated with inflammatory response (e.g., *IL17A, CD14*, and *LTA*). KEGG analysis identified dysregulated biological pathways in bTB PCR-P and bTB PCR-N cattle associated with cell death and phagocytosis, respectively. Previous findings indicated that *M. tuberculosis* inhibits host cell apoptosis as one of its pathogenic mechanisms (25, 26). Taking into account that the level of expression of antiapoptotic factors (e.g., *BCL2* and *BCL2L1*) increased significantly and the level of expression of apoptotic factors (e.g., *TP53INP1* and *TPRKB*) decreased significantly, we hypothesized that, like *M. tuberculosis, M. bovis* inhibits apoptosis. Phagocytosis plays an important role in the immune response against tuberculosis. Interestingly, we found the DEGs associated with phagocytosis were significantly enriched in bTB PCR-N cattle and were associated with biological processes (phagocytosis and engulfment) and pathways (phagosomes and lysosomes). Although phagocytosis efficiently kills mycobacteria, mycobacteria also inhibit phagosome–lysosomes fusion for intracellular survival (27). However, our results indicated that dysregulated phagocytosis was only characteristic of bTB PCR-N cattle, and the thorough studies are needed to reveal the exact underlying mechanisms. Moreover, several dysregulated biological pathways associated with the occurrence and development of tuberculosis were observed in bTB PCR-P and bTB PCR-N cattle. For example, cytokine–cytokine receptor interaction are critical for balancing innate and adaptive immune cells and NF-kappa B signaling pathway is key for regulating immune functions that play important roles in TB progression (28, 29).

Collectively, our current transcriptomic results suggest that bTB PCR-P and bTB PCR-N cattle exhibit various immune responses to *M. bovis*-specific antigen and provided a backbone to leverage our grouping method based on the stages of bTB progression.

Although several biomarkers have been developed for bTB diagnosis (6), disease-specific transcriptional biomarkers are still helpful for bTB control and may provide a supplement or alternative to traditional diagnostic methods, such as TST and IGRAs. Previous studies suggested that LTA serves a protective role during *M. tuberculosis* infection and the IL-8–pathogen binding contributes to the increased mycobactericidal properties of macrophages and neutrophils (30, 31). Here, high *LTA* and *IL-8* concentrations in bTB cattle were observed and the results strongly suggested that *LTA* and *IL-8* can potentially enable differentiation of bTB cattle from healthy cattle (Figures 4A,B). *Mycobacterium tuberculosis* can inhibit host cell apoptosis by inducing *BCL2* over-expression to evade host immune responses (32). We detected high *BCL2* mRNA expression only in bTB PCR-P cattle (Figure 4C). We suspected that apoptosis inhibition acts an important strategy for intracellular *M. bovis* survival during early infection. Given the good performance of *BCL2* in differentiating bTB PCR-P from bTB PCR-N cattle, exactly how *M. bovis* utilizes *BCL2* in host cells needs further study. CHI3L1 plays a significant role in tissue inflammation and remodeling and has been implicated in the pathogenesis of granulomatous and fibrotic interstitial lung diseases (33). To our knowledge, while high levels of CHI3L1 have been observed in many lung diseases (34), only this study assessed this factor as a diagnostic tool for bTB. Granulomas act as protective structures by sealing off and limiting mycobacteria. *CHI3L1* mRNA expression significantly increased in bTB PCR-N cattle (Figure 4D), which may indicate the formation of granuloma. This may explain why the bTB PCR-N cattle did not shed mycobacteria.

In conclusion, our transcriptome data with PBMCs revealed the immune heterogeneity in mycobacteria shedding and non-shedding bTB infected cattle which can improve the understanding of the molecular mechanisms in bTB progression. Importantly, we introduced several promising biomarkers for bTB diagnosis. Given that some mRNAs were highly expressed in mycobacteria shedding or non-shedding bTB infected cattle, their detection may be robustly useful for early diagnosis and eradication of bTB. However, there were some limitations in this study: It was not possible to confirm the infection of *M. bovis* and the stages of bTB progression by slaughtering all cattle used in this study. Further, large-scale clinical test is required to validate these putative biomarkers for bTB diagnosis.

## Data Availability Statement

The datasets generated for this study can be found in NCBI GEO, NCBI Accession No. GSE149505.

## Ethics Statement

The animal study was reviewed and approved by Animal Care and Use Committee of the China Institute of Veterinary Drug Control (SYXK 2005-0021).

## Author Contributions

TX, LJ, and JD designed the experiments. LF, WL, HJ, XGa, XS, YJ, SH, LZ, and XGu performed the experiments. TX, LJ, LF, HZ, and WL analyzed the data. LF, TX, and LJ wrote and revised the paper.

## Conflict of Interest

The authors declare that the research was conducted in the absence of any commercial or financial relationships that could be construed as a potential conflict of interest.

## Abbreviations

AUC: area under the curve
BCL-2: B cell lymphoma 2
bTB: bovine tuberculosis
CHI3L1: chitinase 3-like 1
DEG: differentially expressed gene
GO: gene ontology
IFN-γ: interferon gamma
IGRA: interferon gamma-release assay
IL-8: interleukin-8
KEGG: Kyoto Encyclopedia of Genes and Genomes
LTA: lymphotoxin alpha
PBMC: peripheral blood mononuclear cell
PBS: phosphate-buffered saline
PCR: polymerase chain reaction
PCR-N: PCR-negative
PCR-P: PCR-positive
PPD-B: purified protein derivative of bovine tuberculin
TST: tuberculin skin test.
