# Supplementary material for: Potential Diagnostic Value of the Peripheral Blood Mononuclear Cell Transcriptome From Cattle With Bovine Tuberculosis
Source: Front Vet Sci. 2020 May 27;7:295. doi: 10.3389/fvets.2020.00295 (PMC7266948; doi:10.3389/fvets.2020.00295)
Supplement: Supplementary file 1 [file Table_1.DOC]

**Supplementary Material**

Supplementary Table 1. Primer pairs used for real-time qRT-PCR

| Gene symbol | Gene name | Strand | Sequence (5′-3′) | Amplicon size (bp) |
| --- | --- | --- | --- | --- |
| *TIMP1* | TIMP metallopeptidase inhibitor 1 | Forward | GAGAGGCTACACCAGAGA | 150 |
|  |  | Reverse | ATGACGACATCGGAGTTG |  |
| *TNFAIP2* | TNF alpha induced protein 2 | Forward | GCCACTTACTCCACCTTG | 121 |
|  |  | Reverse | TGTTGATGTCCAGAATGCT |  |
| *THEMIS2* | Thymocyte selection associated family member 2 | Forward | CGCTGGAAGAACCTTACAT | 181 |
|  |  | Reverse | GTAGGAACCTGGAACTGAG |  |
| *LRP1* | LDL receptor related protein 1 | Forward | CCTGGAACCTGTAACCTAC | 103 |
|  |  | Reverse | ACTTGTCGCCTGTGTAAC |  |
| *CYP27B1* | Cytochrome P450 family 27 subfamily B member 1 | Forward | CACTTGCCATAGCCTGAG | 185 |
|  |  | Reverse | CCTTCTGCCACATCATCC |  |
| *PSTPIP2* | Proline-serine-threonine phosphatase interacting protein 2 | Forward | GGCAGTGTGACTATGGAAT | 191 |
|  |  | Reverse | CCTCTACTTCTGTGTCTGTC |  |
| *SAA1* | Serum amyloid A1 | Forward | CATTCTCTGCTCCTTGGT | 122 |
|  |  | Reverse | CCTTGTAGTTGGCTTCTCT |  |
| *CD69* | CD69 molecule | Forward | CATCACCACTCTCATTATAGC | 127 |
|  |  | Reverse | GTGTCCAATCCAATCATCTG |  |
| *IFIT1* | Interferon-induced protein with tetratricopeptide repeats 1 | Forward | TGACTGGCTGTGACTACT | 114 |
|  |  | Reverse | CACTTCGTCTCTGCTGTT |  |
| *IL17F* | Interleukin 17F | Forward | GACTCAGGCACAGAAGGA | 103 |
|  |  | Reverse | AACAGCAGCAGGAACTTG |  |
| *IL17* | Interleukin 17 | Forward | ACCTCACCTTGGACTCTC | 102 |
|  |  | Reverse | GCCTTCAGCATTGATACAG |  |
| *THBS1* | Thrombospondin 1 | Forward | TGAGAGCAGGTAGTTGAGA | 102 |
|  |  | Reverse | ATCATCCAGTCTAAGCACAA |  |
| *AMT* | Aminomethyltransferase | Forward | GGAGAGTCTAGTGGTTGGA | 110 |
|  |  | Reverse | CGCTGGTCACAATCAAGT |  |
| *CLCN7* | Chloride voltage gated channel 7 | Forward | ATTACTGGCTGACGATGTT | 115 |
|  |  | Reverse | GAGTAGATGAGCACGAAGG |  |
|  |  | Reverse | GAGTAGATGAGCACGAAGG |  |
| *LTA* | Lymphotoxin alpha | Forward | GGAATAATAGCGAGACATCAG | 138 |
|  |  | Reverse | TTCACTTCCTTCCAGAACC |  |
| *CHI3L1* | Chitinase 3 like 1 | Forward | GAAGCAGCAACCACACTA | 115 |
|  |  | Reverse | TATTGAGCAGGTCCAGAAC |  |
| *BCL2* | BCL2, apoptosis regulator | Forward | TTCTCAGCGTGTAACTTGT | 113 |
|  |  | Reverse | TCTACCTCCTCCGTGATG |  |
| *IL-8* | Interleukin-8 | Forward | TGAGAAGCAAGATCCATGAA | 120 |
|  |  | Reverse | ATGAGGCACTGAAGTAGATT |  |
| *CRP* | C reactive protein | Forward | GCTGTGGTAGTGAGGTTAG | 153 |
|  |  | Reverse | TGGTGTTGAGGAAGGTAGA |  |
| *BCL2L1* | BCL2 like 1 | Forward | AGTGAGTGAGCAGGTGTT | 165 |
|  |  | Reverse | CTCAGTTCTGTTCTCTTCCA |  |
| *IFNG* | Interferon gamma | Forward | GGATTCTGAGCCACTACTT | 108 |
|  |  | Reverse | CAGGTATAAGGTGAGATGAGA |  |
| *TNF-α* | Tumor necrosis factor | Forward | AGGACTGGAACTGGAACT | 121 |
|  |  | Reverse | AGGAATGTTGCGAAGTGT |  |
| *TLR2* | Toll like receptor 2 | Forward | ATGCTGCCATTCTGATTCT | 104 |
|  |  | Reverse | CCACTCCAGGTAGGTCTT |  |
| *ACTB* | Actin, β | Forward | CGAGATGAGATTGACATTGC | 159 |
|  |  | Reverse | CCACTGCCACATTGTAGAA |  |
