# Supplementary material for: Potential Diagnostic Value of the Peripheral Blood Mononuclear Cell Transcriptome From Cattle With Bovine Tuberculosis
Source: Front Vet Sci. 2020 May 27;7:295. doi: 10.3389/fvets.2020.00295 (PMC7266948; doi:10.3389/fvets.2020.00295)
Supplement: Supplementary file 5 [file Table_5.DOC]

**Supplementary Material**

Supplementary Table 5. The list of transcriptional gene signatures.

| Gene name | Summary ( provided by RefSeq) |
| --- | --- |
| *IFNG* (Interferon gamma) | The encoded protein is related to viral, bacterial and parasitic infections and to several autoimmune diseases. |
| *TNF* (tumor necrosis factor) | The cytokine is involved in the regulation of a wide spectrum of biological processes including cell proliferation, differentiation, apoptosis, lipid metabolism, and coagulation. |
| *IL8* (Interleukin-8) | The encoded protein is secreted primarily by neutrophils, where it serves as a chemotactic factor by guiding the neutrophils to the site of infection. |
| *CRP* (C reactive protein) | The encoded protein is involved in several host defense related functions based on its ability to recognize foreign pathogens and damaged cells of the host and to initiate their elimination by interacting with humoral and cellular effector systems in the blood. |
| *BCL2L1* (BCL2 like 1) | The encoded protein belongs to the BCL-2 protein family. BCL-2 family members form hetero- or homodimers and act as anti- or pro-apoptotic regulators that are involved in a wide variety of cellular activities. |
| *BCL2* (BCL2, apoptosis regulator) | This gene encodes an integral outer mitochondrial membrane protein that blocks the apoptotic death of some cells such as lymphocytes. |
| *TLR2* (Toll like receptor 2 ) | The encoded protein is a member of the Toll-like receptor (TLR) family which plays a fundamental role in pathogen recognition and activation of innate immunity. |
| *CHI3L1* (Chitinase 3 like 1) | The encoded protein is thought to play a role in the process of inflammation and tissue remodeling. |
| *LTA* (Lymphotoxin alpha) | The encoded protein mediates a large variety of inflammatory, immunostimulatory, and antiviral responses, is involved in the formation of secondary lymphoid organs during development and plays a role in apoptosis. |
