# Supplementary material for: Potential Diagnostic Value of the Peripheral Blood Mononuclear Cell Transcriptome From Cattle With Bovine Tuberculosis
Source: Front Vet Sci. 2020 May 27;7:295. doi: 10.3389/fvets.2020.00295 (PMC7266948; doi:10.3389/fvets.2020.00295)
Supplement: Supplementary file 6 [file Image_1.PDF]

Supplementary Material

Supplemental Figures

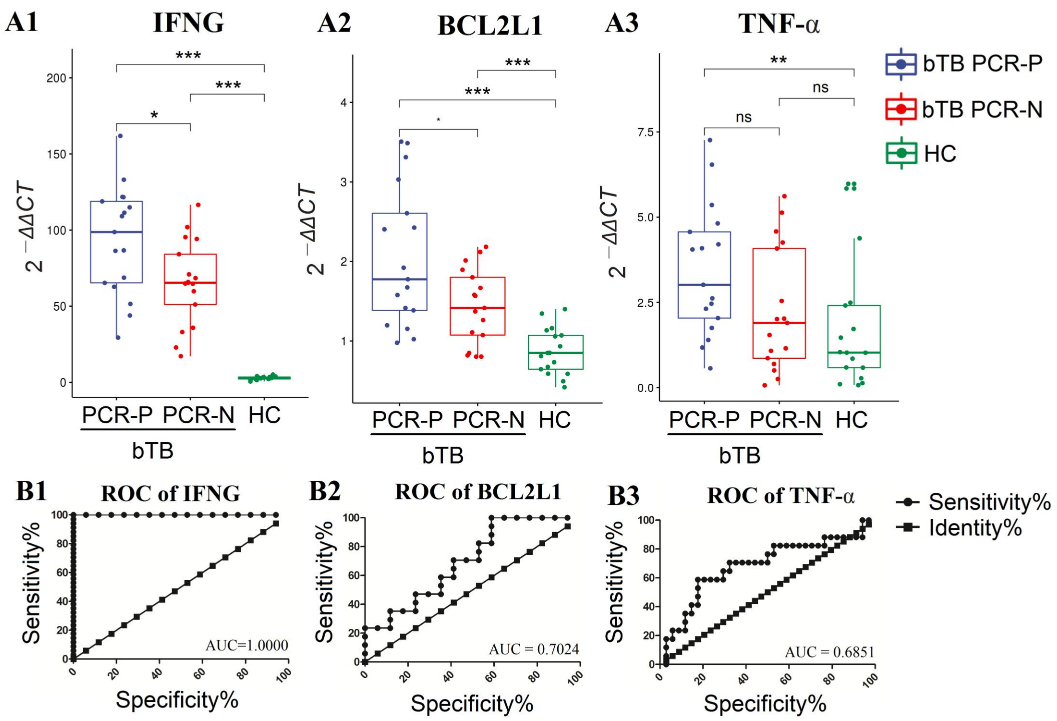

Figure S1. Validation the performance of PBMCs transcripts. (**A1-3**) The levels of PBMCs transcripts expression in bTB PCR-P (n=17) ,bTB PCR-N (n=17) and HC cattle (n=17). Expression level of *IFNG* (**A1**), *BCL2L1*(**A2**), and *TNF-α* (A3). Gene expression was calculated using the $2^{-\Delta\Delta CT}$ method, with unstimulated PBMCs as a calibrator and β-actin as the endogenous control. Dots indicate individual data points, box limits show the 25th and 75th percentiles, center lines indicate the medians, and whiskers extend 1.5 times the interquartile range (IQR). Significant differences were tested using a one-way ANOVA followed by Dunn's multiple-comparison test. ***, $P < 0.0001$; **, $P < 0.001$; *, $P < 0.05$. (**B1-3**)The diagnostic values of *IFNG* (**B1**), *BCL2L1* (**B2**), and *TNF-α* (**B3**) were analyzed by receiver operating characteristic (ROC) curves.
